# Evolution of Management and Outcomes of Infective Endocarditis After Introducing the Endocarditis Team

**DOI:** 10.1016/j.jacadv.2025.102579

**Published:** 2026-01-29

**Authors:** Eefje M. Dalebout, Ricardo P.J. Budde, Tjebbe W. Galema, Ali R. Wahadat, Laurens E. Swart, Kevin M. Veen, Nelianne J. Verkaik, Alexander Hirsch, Jolanda Kluin, Jolien W. Roos-Hesselink

**Affiliations:** a Department of Cardiology, Cardiovascular Institute, Thorax Center, Erasmus Medical Center, Rotterdam, the Netherlands; b Department of Radiology and Nuclear Medicine, Erasmus Medical Center, Rotterdam, the Netherlands; c Department of Cardiothoracic Surgery, Thorax Center, Erasmus Medical Center, Rotterdam, the Netherlands; d Department of Cardiology, Spaarne Gasthuis, Haarlem, the Netherlands; e Department of Medical Microbiology and Infectious Diseases, Erasmus MC, Rotterdam, The Netherlands

**Keywords:** endocarditis, endocarditis team, multidisciplinary care, valvular heart disease

## Abstract

**Background:**

Current guidelines recommend discussing patients with infective endocarditis in an endocarditis team (ET). The benefits of an ET have been established, but not the evolution over time.

**Objectives:**

The objective of the study was to investigate changes in management and outcomes in infective endocarditis over time.

**Methods:**

All patients discussed by our ET since 2016 were prospectively included and divided into 3 cohorts. Trends over time for the number of discussions, diagnostics, ET recommendations, treatment, and outcomes were assessed using Poisson, logistic, and Cox regression, respectively, adjusted for potential case mix.

**Results:**

The total cohort included 1,042 patients, with 223, 364, and 455 patients in cohorts 1 (2016-2018), 2 (2019-2021), and 3 (2022-2024), respectively. The percentage of patients with a rediscussion increased with 18.2% (*P* < 0.001), and the number of discussions per patient increased with 3% per diagnosis-year (*P* = 0.006). The percentage of patients with positive blood cultures decreased with 17.5% (*P* < 0.001), whereas cardiac computed tomography (CT) and positron emission tomography (PET) CT usage increased with 3.1% (*P* = 0.028) and 12.5% (*P* < 0.001), respectively. Advice for additional diagnostic tests decreased by 11.6% (*P* < 0.001), and treatment change decreased by 18.3% (*P* < 0.001). Reclassification of the initial diagnosis increased with 8.3% (*P* = 0.045). Relapse (*P* = 0.82) and reinfection rates did not change (*P* = 0.98). One-year survival was approximately 80% and remained stable (*P* = 0.62).

**Conclusions:**

There were more referrals and rediscussions, but less need for additional advice, reflecting increased familiarity with and a learning effect of the ET. CT and PET-CT were used more often. Mortality did not change over time and remained high.

The age-standardized incidence rate for infective endocarditis (IE) is approximately 14 per 100,000 person-years globally.^1^ The diagnosis is often missed due to nonspecific symptoms and IE is known for its high morbidity and mortality.^1^^,^^2^ To improve outcomes, the 2015 European Society of Cardiology (ESC) guidelines for the management of endocarditis recommend discussion of patients with (suspected) IE in a multidisciplinary endocarditis team (ET).^3^ Studies on the effect of the ET have shown benefits for patient management and outcomes, thus other international guidelines have adopted this advice.^4^, ^5^, ^6^, ^7^ Although studies have shown a positive impact of having an ET on patient management and outcomes in the first years after implementation of the ET, development of the ET in the years after its introduction has not been described.^6^^,^^7^ The aim of this study was to assess the evolution of the ET in diagnosis, management, and outcomes of patients with (suspected) IE in the 9 years after its installation at our institution.

## Methods

### Study design

The ET was installed at our institution at the end of 2015, and consists of at least 1 cardiologist, clinical microbiologist or infectious diseases specialist, radiologist and/or nuclear medicine physician, and cardiothoracic surgeon from Erasmus MC. In addition, attendance of the referring doctor and clinical microbiologist of the referring center is preferred. Originally, discussions were held when needed. From the beginning of 2016, as more regional centers in our catchment area referred their patients to our tertiary center, they became weekly and now even biweekly meetings.^8^ All patients were prospectively included in a database and information was recorded during the ET discussion. All adult patients discussed in the ET from January 2016 until January 2025 are included in this prospective cohort study. The local Medical Research Ethics Committee approved the study with a waiver for the need for informed consent (MEC-2024-0125).

### Data collection and definitions

Data were collected from the prospectively registered, standardized ET documentation and electronic patient records. The included patients were divided into 3 cohorts evenly spread across the 9 years. Cohorts 1, 2, and 3 consist of patients first discussed in the period 2016 to 2018, 2019 to 2021, and 2022 to 2024, respectively. The final diagnosis consisted of definite, possible, or rejected IE and was defined as a consensus diagnosis made by the ET after (re)discussing the patient and based on the ESC guidelines that were applicable at that time.^3^^,^^5^ Suspected infection location comprised native valve endocarditis (NVE), prosthetic valve endocarditis (PVE), transcatheter aortic valve (TAVI) IE, cardiac implantable electronic device infection, vascular prosthesis infection, and “other” infected sites like stents, atrial septal defect-closure devices, patches, and abandoned leads. The PVE group included patients with surgical prosthetic valves, valve repair (plasty), and percutaneously implanted prosthetic valves (excluding TAVI). At presentation, more than 1 site could be suspected of IE. In the possible/definite IE group, only 1 main location was depicted, following a hierarchical categorization if needed: TAVI-IE, PVE, cardiac implantable electronic device infection, vascular graft infection, NVE, or other.

Relapse was defined as a new episode of IE after completing antibiotic therapy, but within 6 months after the initial episode, and reinfection as a new episode of IE, typically more than 6 months after the initial episode, or IE with a different microorganism. The indication for surgery or intervention was defined in accordance with the 2015 and 2023 ESC guidelines for the management of endocarditis.^3^^,^^5^

The follow-up time was defined as the time between the first discussion and the date the electronic patient file was last checked for relapse, reinfection, or survival. Data about all-cause mortality were derived from the Dutch national population database. Survival data were checked for all patients on January 6, 2025. The COVID-19 period was defined as March 2020-March 2022.

### Statistics

Categorical variables are reported as frequency (proportion) and compared using the chi-square test or Fisher exact test, as appropriate. Continuous data are reported using mean ± SD (Gaussian distribution) or medians and 25th-75th percentiles [Q1-Q3] (non-Gaussian distribution), and compared using analysis of variance or Kruskal-Wallis test, as appropriate. Baseline characteristics are stratified into 3 groups. Survival probability was estimated using the Kaplan-Meier estimator and subgroup comparisons were made by log-rank test. Patients were censored at the last date with information on relapse, reinfection, or survival status. Missing data were handled using complete-case analyses (Supplemental Table 1). Follow-up completeness for survival was assessed using the Modified Clark C method.^9^

### Trends over time

To examine trends over time, logistic regression was used for binary variables, Poisson models for count variables, and Cox regression for survival data (survival censored at 1-year postdiagnosis or at the last date of follow-up if this was within 1 year, and survival for the complete follow-up time). The results of the logistic, Poisson, and Cox regression models are presented as adjusted odds ratio (aOR) with 95% confidence interval (CI), adjusted incidence rate ratio (aIRR) with 95% CI, or adjusted hazard ratio (aHR) with 95% CI, respectively. All models were adjusted for baseline characteristics, including IE type (native valve or prosthetic) and the COVID-19 period. Covariates were selected based on clinical relevance, with a full model applied to prioritize inference over prediction. Correlation between the variables was tested by Spearman’s correlation coefficient, to identify potential multicollinearity. Year of diagnosis was modeled linearly in the logistic and Poisson models, whereas in the Cox model for 1-year survival, splines with 2 knots allowed for more flexibility. The proportional hazards assumption was tested using Schoenfeld residuals, and if violated, the Cox model was stratified for those variables. Multicollinearity was checked using variance inflation factor and linearity was checked using partial residuals plots (logistic and Poisson regression) and martingale residual plots (Cox regression). In case of overdispersion in the Poisson models, a quasipoisson model was conducted. Cumulative incidence curves for relapse and reinfection were conducted using a nonparametric cumulative incidence estimator function with death as a competing risk. Analyses were performed using R (version 4.4.2; R core team).^10^ A *P* value <0.05 was considered statistically significant.

## Results

**Figure 1:**
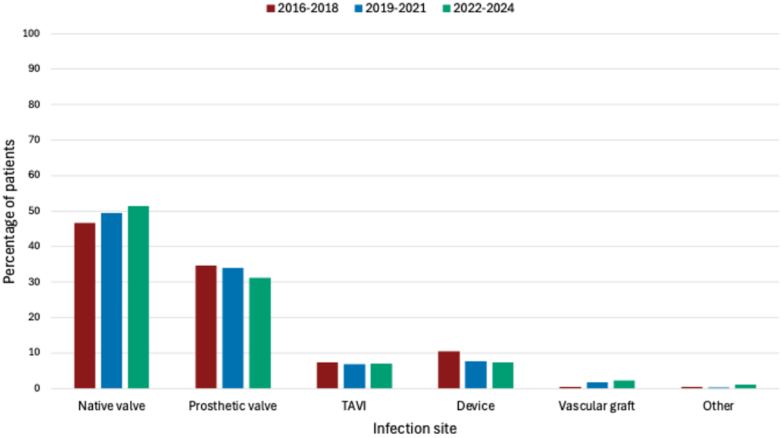
Infection Location in Patients With Possible/Definite Infective Endocarditis This figure shows the percentage of patients (y-axis) with definite/possible infective endocarditis with native valve endocarditis, prosthetic valve endocarditis, transcatheter aortic valve endocarditis, cardiac implantable electronic device infection, vascular graft infection, or other cardiac sites or devices involved. Only 1 location can be present within 1 patient. Cohort 1 (2016-2018) is shown in red, cohort 2 (2019-2021) in blue, and cohort 3 (2022-2024) in green. All *P* values >0.05 (chi-squared). TAVI = transcatheter aortic valve.

**Figure 2:**
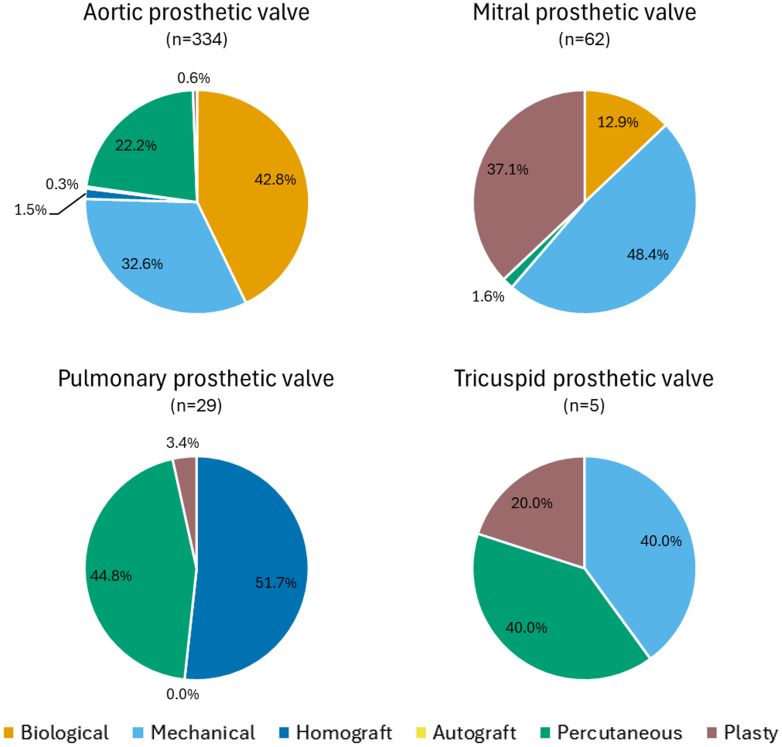
Type and Location of Prosthetic Valves Suspected for Infection This figure shows the location (aortic, mitral, pulmonary, or tricuspid) of prosthetic valves suspected for infection for the total cohort, with the percentage of prosthetic valve types per valve location. “n” indicates the number of patients per group. Within 1 patient, multiple locations of infection can be present.

In the study period, 1,042 individual patients were discussed in the ET, with 223, 364, and 455 patients in cohort 1, 2, and 3, respectively. Overall, 41.4% of patients were discussed multiple times, increasing from 26.0% in cohort 1 to 47.3% in cohort 2, and 44.2% in cohort 3 (*P* < 0.001). Table 1 provides an overview of baseline characteristics. Figures 1 and 2, and Supplemental Tables 2 and 3, provide detailed information on the infection location. The average discussion frequencies were 1.4, 1.8, and 1.7 for cohort 1, 2, and 3, respectively. Poisson regression model showed a significant increase in the number of discussions per patient of 3% per year (aIRR: 1.03; 95% CI: 1.01-1.05; *P* = 0.006).

**Table 1:** Baseline Characteristics of the Different Cohorts

	Total (N = 1,042)	2016-2018 (n = 223)	2019-2021 (n = 364)	2022-2024 (n = 455)	*P* Value
Referred from other hospital	758 (72.7)	158 (70.9)	262 (72.0)	338 (74.3)	0.59
Age (y)	67 [56-76]	67 [55-76]	67 [54-76]	68 [58-76]	0.72
Sex					0.98
Male	745 (71.5)	160 (71.7)	259 (71.2)	326 (71.6)	
Female	297 (28.5)	63 (28.3)	105 (28.8)	129 (28.4)	
Prior episode of endocarditis	62 (6.0)	17 (7.6)	31 (8.5)	14 (3.1)	0.002
Pre-existent valve disease	490 (47.0)	120 (53.8)	183 (50.3)	187 (41.1)	0.002
Congenital heart disease	140 (13.4)	24 (10.8)	47 (12.9)	69 (15.2)	0.27
Prior heart valve surgery	429 (41.2)	96 (43.0)	155 (42.5)	178 (39.1)	0.49
Prior device implantation	184 (17.7)	52 (23.3)	62 (17.0)	70 (15.4)	0.036
Type of prior device implantation					0.039
Pacemaker^a^	108 (58.7)	32 (61.5)	37 (59.7)	39 (55.7)	
ICD^a^	54 (29.3)	19 (36.5)	16 (25.8)	19 (27.1)	
CRTP^a^	3 (1.6)	1 (1.9)	1 (1.6)	1 (1.4)	
CRTD^a^	19 (10.3)	0 (0.0)	8 (12.9)	11 (15.7)	
Prior vascular prosthesis	120 (11.5)	21 (9.4)	36 (9.9)	63 (13.8)	0.12
Type of prior vascular prosthesis					<0.001
Ascending aorta^a^	14 (11.7)	0 (0.0)	9 (25.0)	5 (7.9)	
Ascending aorta + other^a^^b^	15 (12.5)	3 (14.3)	2 (5.6)	10 (15.9)	
Bentall^a^	69 (57.5)	18 (85.7)	22 (61.1)	29 (46.0)	
Bentall + other^a^^b^	5 (4.2)	0 (0.0)	2 (5.6)	3 (4.8)	
Other^a^^b^	17 (14.2)	0 (0.0)	1 (2.8)	16 (25.4)	
Hypertension	379 (36.4)	57 (25.6)	142 (39.0)	180 (39.6)	0.001
Heart failure	154 (14.8)	32 (14.3)	61 (16.8)	61 (13.4)	0.40
Chronic renal failure	106 (10.2)	23 (10.3)	44 (12.1)	39 (8.6)	0.25
CVA	158 (15.2)	30 (13.5)	57 (15.7)	71 (15.6)	0.72
COPD	82 (7.9)	20 (9.0)	29 (8.0)	33 (7.3)	0.74
Diabetes mellitus	188 (18.0)	41 (18.4)	63 (17.3)	84 (18.5)	0.90
Intravenous drug use^c^	6 (0.6)	1 (0.1)	1 (0.1)	4 (0.4)	0.51

Data are presented as n (%) or median [25th-75th percentiles]. “n” describes the number of patients in the subgroup.

COPD = chronic obstructive pulmonary disease; CRTD = cardiac resynchronization therapy with a defibrillator; CRTP = Cardiac resynchronization therapy with a pacemaker; CVA = cerebrovascular accident; ICD = implantable cardioverter defibrillator.

a Percentage within group.

b Other vascular graft (eg, hemi-arch, thoracic endovascular aortic repair).

c Within the subgroup of patients with right-sided or cardiac implantable electronic device infection.

### Diagnostic criteria

In total, 74.6% had 2 or more positive blood cultures, meeting 1 of the major criteria. This significantly decreased from 87.0% in cohort 1, to 73.4% in cohort 2, and 69.5% in cohort 3 (aOR: 0.85; 95% CI: 0.80-0.91; *P* < 0.001). Among patients with possible/definite IE, the percentage of patients with positive blood cultures also decreased (cohort 1: 92.7%, cohort 2: 80.2%, cohort 3 78.1% [aOR: 0.82; 95% CI: 0.75-0.90; *P* < 0.001]). The percentage of patients with possible/definite IE without any positive blood culture was not significantly different (cohort 1: 4.7%, cohort 2: 8.6%, cohort 3: 4.1% [aOR: 0.96; 95% CI: 0.84-1.09; *P* = 0.49]). The most common causative pathogens were *Staphylococcus aureus* (25.5%), *Viridans* group *streptococci* (21.8%), and *Enterococcus* spp. (13.4%), which did not change over time (*P* = 0.85). Table 2 shows causative pathogens for patients with possible/definite IE.

**Table 2:** Causative Pathogen for Different Infection Locations in Patients With Possible/Definite Infective Endocarditis

Causative Pathogen	Total (N = 864)	Native Valve (n = 429)	Prosthetic Valve (n = 284)	TAVI (n = 61)	Device (n = 70)	Vascular Prosthesis (n = 14)	Other (n = 6)	*P* Value
*S aureus*	220 (25.5)	112 (26.1)	53 (18.7)	10 (16.4)	38 (54.3)	5 (35.7)	2 (33.3)	<0.001
*Viridans* group streptococci	188 (21.8)	128 (29.8)	43 (15.1)	13 (21.3)	2 (2.9)	1 (7.1)	1 (16.7)	<0.001
*E faecalis*/*faecium*	116 (13.4)	45 (10.5)	39 (13.7)	19 (31.1)	10 (14.3)	2 (14.3)	1 (16.7)	0.003
*S bovis*	56 (6.5)	27 (6.3)	22 (7.7)	5 (8.2)	2 (2.9)	0 (0.0)	0 (0.0)	0.67
Other streptococci	52 (6.0)	33 (7.7)	13 (4.6)	1 (1.6)	3 (4.3)	1 (7.1)	1 (16.7)	0.16
CoNS	49 (5.7)	16 (3.7)	17 (6.0)	6 (9.8)	9 (12.9)	0 (0.0)	1 (16.7)	0.015
Cutibacterium spp	38 (4.4)	0 (0.0)	36 (12.7)	0 (0.0)	1 (1.4)	1 (7.1)	0 (0.0)	<0.001
HACEK organisms	16 (1.9)	7 (1.6)	6 (2.1)	0 (0.0)	3 (4.3)	0 (0.0)	0 (0.0)	0.53
*S anginosus*	11 (1.3)	9 (2.1)	2 (0.7)	0 (0.0)	0 (0.0)	0 (0.0)	0 (0.0)	0.50
*S pneumoniae*	8 (0.9)	7 (1.6)	1 (0.4)	0 (0.0)	0 (0.0)	0 (0.0)	0 (0.0)	0.48
*S lugdunensis*	5 (0.6)	2 (0.5)	3 (1.1)	0 (0.0)	0 (0.0)	0 (0.0)	0 (0.0)	0.77
*E coli*	3 (0.3)	1 (0.2)	1 (0.4)	0 (0.0)	1 (1.4)	0 (0.0)	0 (0.0)	0.47
Other pathogen	52 (6.0)	19 (4.4)	27 (9.5)	4 (6.6)	0 (0.0)	2 (14.3)	0 (0.0)	0.006
Culture negative	50 (5.8)	23 (5.4)	21 (7.4)	3 (4.9)	1 (1.4)	2 (14.3)	0 (0.0)	0.25

Data are presented as n (%). “n” describes the number of patients in the subgroup.

CoNS = Coagulase negative staphylococci; HACEK = *Haemophilus, Aggregatibacter, Cardiobacterium, Eikenella, Kingella*; TAVI = transcatheter aortic valve.

**Figure 3:**
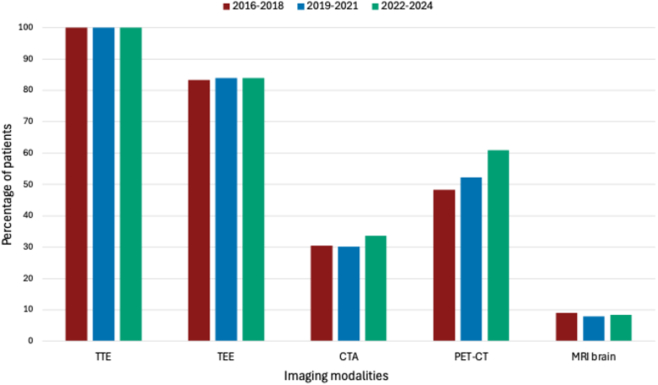
Diagnostic Imaging Modalities Used in Each Cohort This figure shows the percentage of patients (y-axis) with TTE, TEE, CTA, PET-CT, and MRI of the brain performed for diagnosis of infective endocarditis in each of the 3 cohorts. Cohort 1 (2016-2018) is shown in red, cohort 2 (2019-2021) in blue, and cohort 3 (2022-2024) in green. Only the use of PET-CT significantly increased over time (*P* value chi-square = 0.003). CTA = computed tomography angiography; MRI = magnetic resonance imaging; PET-CT = positron emission tomography and computed tomography; TEE = transesophageal echocardiography; TTE = transthoracic echocardiography.

Overall, imaging was positive in 77.7% and not different between the cohorts (aOR: 0.95; 95% CI: 0.89-1.01; *P* = 0.11). The use of different imaging modalities and their results are shown in Table 3 and Figure 3. Although the use of transthoracic echocardiography and transesophageal echocardiography (TEE) did not differ between the cohorts, the use of computed tomography (CT) angiography (CTA) (cohort 1: 30.5%, cohort 2: 30.2%, cohort 3: 33.6% [aOR: 1.07; 95% CI: 1.01-1.13; *P* = 0.028]) and positron emission tomography CT (PET-CT) increased (cohort 1: 48.4%, cohort 2: 52.2%, cohort 3: 60.9% [aOR: 1.12; 95% CI: 1.06-1.18; *P* < 0.001]). Subgroup analysis showed that the use of PET-CT in patients with suspected NVE increased (cohort 1: 27.6%, cohort 2: 35.7%, cohort 3: 45.6% [aOR: 1.13; 95% CI: 1.04-1.22; *P* = 0.002]), but not in patients suspected with PVE (cohort 1: 66.7%, cohort 2: 65.0%, cohort 3: 70.0% [aOR: 1.02; 95% CI: 0.93-1.12; *P* = 0.60]). The proportion of positive CTA and PET-CT scans remained the same, with 62.2% (aOR: 0.92; 95% CI: 0.84-1.01; *P* = 0.098) and 47.5% (aOR: 1.03; 95% CI: 0.95-1.11; *P* = 0.45) positive scans overall, respectively.

**Table 3:** Diagnostic Criteria, Tests, Advice and Final Diagnosis by the Endocarditis Team in the Different Cohorts

	Total (N = 1,042)	2016-2018 (n = 223)	2019-2021 (n = 364)	2022-2024 (n = 455)	aOR Time (95% CI)	*P* Value^c^
Major diagnostic criteria						
Positive blood cultures	777 (74.6)	194 (87.0)	267 (73.4)	316 (69.5)	0.85 (0.80-0.91)	<0.001
Positive imaging	810 (77.7)	175 (78.5)	292 (80.2)	343 (75.4)	0.95 (0.89-1.01)	0.11
Minor diagnostic criteria						
Predisposition	733 (70.3)	171 (76.7)	258 (70.9)	304 (66.8)	0.76 (0.68-0.85)	<0.001
Fever	716 (68.8)	157 (70.7)	237 (65.1)	322 (70.8)	1.03 (0.97-1.09)	0.33
Embolic complications	389 (37.3)	82 (36.8)	126 (34.6)	181 (39.8)	1.02 (0.96-1.07)	0.57
Cerebral embolization^a^	133 (34.2)	32 (39.0)	46 (36.5)	55 (30.3)	0.95 (0.87-1.04)	0.24
Cerebral embolization at presentation^a^	102 (76.7)	28 (87.5)	31 (67.4)	42 (78.2)	1.10 (1.00-1.22)	0.046
Other embolic complication^a^	289 (74.3)	55 (67.1)	88 (69.8)	146 (80.7)	0.94 (0.77-1.16)	0.59
Other microbiological evidence^b^	146 (14.0)	7 (3.1)	46 (12.6)	93 (20.4)	1.34 (1.22-1.49)	<0.001
Immunological phenomena	18 (1.7)	3 (1.3)	9 (2.5)	6 (1.3)	0.95 (0.74-1.22)	0.68
Imaging tests						
TTE/TEE performed	1,042 (100.0)	223 (100.0)	364 (100.0)	455 (100.0)	n.a.	n.a.
TTE/TEE positive	676 (65.0)	144 (64.6)	244 (67.4)	288 (63.3)	0.97 (0.92-1.03)	0.40
TEE performed	874 (83.9)	186 (83.4)	306 (84.1)	382 (84.0)	0.99 (0.93-1.07)	0.88
TEE positive^a^	599 (68.5)	126 (67.7)	214 (79.9)	259 (67.8)	0.99 (0.93-1.05)	0.78
CTA performed	331 (31.8)	68 (30.5)	110 (30.2)	153 (33.6)	1.07 (1.01-1.13)	0.028
CTA positive^a^	206 (62.2)	48 (70.6)	63 (57.3)	95 (62.1)	0.92 (0.84-1.01)	0.098
PET-CT performed	575 (55.2)	108 (48.4)	190 (52.2)	277 (60.9)	1.12 (1.06-1.18)	<0.001
PET-CT positive^a^	273 (47.5)	54 (50.0)	90 (47.4)	129 (46.6)	1.03 (0.95-1.11)	0.45
Brain MRI performed	87 (8.3)	20 (9.0)	29 (8.0)	38 (8.4)	0.97 (0.89-1.06)	0.52
Brain MRI positive^a^	67 (77.0)	15 (75.0)	22 (75.9)	30 (78.9)	1.19 (0.92-1.56)	0.19
Advice of the endocarditis team						
Additional diagnostic test	481 (46.2)	116 (52.0)	181 (49.7)	184 (40.4)	0.90 (0.86-0.95)	<0.001
Microbiological^a^	136 (28.3)	42 (36.2)	62 (34.3)	32 (17.4)	0.84 (0.77-0.92)	<0.001
Imaging^a^	348 (72.3)	79 (68.1)	140 (77.3)	129 (70.1)	1.01 (0.93-1.10)	0.76
Other^a^	102 (21.2)	17 (14.7)	30 (16.6)	55 (29.9)	1.18 (1.07-1.30)	0.001
Additional test changed therapy	81 (16.9)	17 (14.3)	36 (19.9)	28 (15.7)	0.98 (0.88-1.08)	0.64
Conservative to invasive^a^	35 (43.2)	8 (47.1)	14 (38.9)	13 (46.4)	0.98 (0.78-1.23)	0.85
Change in antibiotic therapy^a^	61 (75.3)	11 (64.7)	31 (86.1)	19 (67.9)	0.85 (0.63-1.13)	0.29
Additional test changed diagnosis	102 (21.5)	13 (11.2)	49 (27.4)	40 (22.3)	1.03 (0.94-1.14)	0.50
Change in treatment (overall)	548 (52.6)	139 (62.3)	209 (57.4)	200 (44.0)	0.86 (0.82-0.91)	<0.001
Change in antibiotic therapy^a^	420 (40.3)	97 (43.5)	165 (45.3)	158 (34.7)	0.93 (0.88-0.98)	0.005
Change in diagnosis (overall)	210 (20.2)	30 (13.5)	81 (22.3)	99 (21.8)	1.07 (1.00-1.14)	0.045
Final diagnosis						
Definite	741 (71.1)	172 (77.1)	258 (70.9)	311 (68.4)	0.93 (0.88-0.98)	0.011
Possible	123 (11.8)	19 (8.5)	45 (12.4)	59 (13.0)	1.09 (1.00-1.18)	0.047
Rejected	178 (17.1)	32 (14.3)	61 (16.8)	85 (18.7)	1.05 (0.98-1.12)	0.18
**Disease characteristics in patients with possible/definite IE**	**(N = 864)**	**(N = 191)**	**(N = 303)**	**(N = 370)**		
Vegetation						
No vegetation	213 (42.3)	47 (42.3)	78 (45.6)	88 (39.6)	0.99 (0.91-1.08)	0.82
≤1 cm	125 (24.8)	27 (24.3)	47 (27.5)	51 (23.0)	0.94 (0.86-1.02)	0.15
>1 cm, ≤1.5 cm	88 (17.5)	15 (13.5)	25 (14.6)	48 (21.6)	1.12 (1.01-1.24)	0.034
>1.5 cm, ≤3 cm	70 (13.9)	20 (18.0)	18 (10.5)	32 (14.4)	0.98 (0.89-1.09)	0.75
>3 cm	8 (1.6)	2 (1.8)	3 (1.8)	3 (1.4)	0.98 (0.74-1.31)	0.88
Severe valve regurgitation	221 (25.6)	52 (27.2)	77 (25.4)	92 (24.9)	0.98 (0.91-1.05)	0.58
Heart failure	104 (12.0)	24 (12.6)	38 (12.5)	42 (11.4)	0.98 (0.90-1.08)	0.72
Periannular complications	151 (17.5)	29 (15.2)	62 (20.5)	60 (16.2)	1.02 (0.94-1.10)	0.63

Data are presented as n (%). “n” describes the number of patients in the subgroup. All models were adjusted for baseline characteristics, including infective endocarditis type (native valve or prosthetic) and the COVID-19 period. The results of the logistic regression analyses for change over time (diagnosis year) are shown as adjusted OR (aOR) with 95% CI, and corresponding *P* value.

CTA = computed tomography angiography; MRI = magnetic resonance imaging; PET-CT = positron emission tomography and computed tomography; TEE = transesophageal echocardiography; TTE = transthoracic echocardiography.

a Percentage within group.

b Other microbiological evidence than major criterion, compliant with the modified dukes criteria.^5^

c *P* value for diagnosis year analyzed in the logistic regression model.

The percentage of patients with a predisposing factor decreased from 76.7% in cohort 1 to 70.9% in cohort 2 and 66.8% in cohort 3 (aOR: 0.76; 95% CI: 0.68-0.85; *P* < 0.001). Fever, embolic complications, immunological phenomena, and other microbiological evidence not meeting the requirements for a major criterion were present in 68.8%, 37.3%, 1.7%, and 14.0% of the total cohort, respectively.

### Advice given by the endocarditis team

The ET advised less additional diagnostic tests over time (cohort 1: 52.0%, cohort 2: 49.7%, cohort 3: 40.4% [aOR: 0.90; 95% CI: 0.86-0.95; *P* < 0.001]). Among the patients with additional diagnostics tests advised, advice for microbiological tests decreased (cohort 1: 36.2%, cohort 2: 34.3%, cohort 3: 17.4%, [aOR: 0.84; 95% CI: 0.77-0.92; *P* < 0.001]), whereas advice for other tests, such as colonoscopy or portal of entry detection, increased (cohort 1: 14.7%, cohort 2: 16.6%, cohort 3: 29.9%, [aOR: 1.18; 95% CI: 1.07-1.30; *P* = 0.001]). Among patients with additional tests advised, the additional tests resulted in a change of treatment in 16.9% and change in diagnosis in 21.5%, and both did not significantly change over time (aOR treatment change after additional testing: 0.98; 95% CI: 0.88-1.08; *P* = 0.64; aOR diagnosis change after additional testing: 1.03; 95% CI: 0.94-1.14; *P* = 0.50). Overall, advice for change in treatment was given in 52.6% and decreased significantly over time (cohort 1: 62.3%, cohort 2: 57.4%, cohort 3: 44.0%, [aOR: 0.86; 95% CI: 0.82-0.91; *P* < 0.001]). The percentage of patients with a change in diagnosis increased (cohort 1: 13.5%, cohort 2: 22.3%, cohort 3: 21.8%, [aOR: 1.07; 95% CI: 1.00-1.14; *P* = 0.045]). After ET discussion, the final diagnosis was definite in 741 (71.1%), possible in 123 (11.8%), and rejected in 178 (17.1%) patients (aOR definite IE: 0.93; 95% CI: 0.88-0.98; *P* = 0.011, aOR possible IE: 1.09; 95% CI: 1.00-1.18; *P* = 0.047, aOR rejected IE: 1.05; 95% CI: 0.98-1.12; *P* = 0.18) (Table 3). Diagnosis changed from possible to definite IE in 53 patients, and from possible to rejected IE in 156 patients. In 1 patient, the prior definite IE diagnosis was changed by the endocarditis team after re-evaluation of the initial TEE after an advised follow-up TEE, and diagnosis changed into possible IE.

### Treatment

The percentage of patients with possible/definite IE treated with a 6-week course of antibiotics remained stable (cohort 1: 88.2%, cohort 2: 83.0%, cohort 3: 86.3% [aOR: 0.99; 95% CI: 0.91-1.08; *P* = 0.85]) (Table 4), as well as the percentage of patients treated with a 4-week course of antibiotics (cohort 1: 12.2%, cohort 2: 17.0%, cohort 3: 13.7% [aOR: 1.01; 95% CI: 0.92-1.08; *P* = 0.85]). Surgery was indicated in 53.5% of the patients; however, surgery was performed in only 39.8% and 60.2% of the patients were treated conservatively. The percentage of patients treated conservatively despite having an indication for surgery increased (cohort 1: 6.8%, cohort 2: 14.2%, cohort 3: 16.8% [aOR: 1.17; 95% CI: 1.07-1.29; *P* < 0.001]) (Table 4). The indication for urgent surgery was not different between the cohorts (all *P* > 0.05, Supplemental Table 4).

**Table 4:** Treatment of the Patients With Possible/Definite Infective Endocarditis

Treatment	Total (N = 864)	2016-2018 (n = 191)	2019-2021 (n = 303)	2022-2024 (n = 370)	aOR Time (95% CI)	*P* Value^b^
Antimicrobial treatment						
6 weeks	689 (85.6)	165 (88.2)	235 (83.0)	289 (86.3)	0.99 (0.91-1.08)	0.85
4 weeks	117 (14.5)	23 (12.2)	48 (17.0)	46 (13.7)	1.01 (0.92-1.08)	0.85
Chronic suppressive therapy	23 (2.7)	6 (3.1)	5 (1.7)	12 (3.2)	1.09 (0.90-1.33)	0.41
Indicated treatment						
Conservative treatment	402 (46.5)	102 (53.4)	138 (45.5)	162 (43.8)	0.94 (0.88-0.99)	0.023
Surgery	462 (53.5)	89 (46.6)	165 (54.5)	208 (56.2)	1.07 (1.01-1.13)	0.023
Elective^a^	80 (17.3)	17 (19.1)	25 (15.2)	38 (18.3)	1.05 (0.95-1.16)	0.37
Urgent^a^	312 (67.5)	59 (66.3)	116 (70.3)	137 (65.9)	1.05 (0.99-1.12)	0.11
Device extraction^a^	59 (12.8)	12 (13.5)	21 (12.7)	26 (12.5)	0.99 (0.86-1.15)	0.90
Urgent + device extraction^a^	11 (2.4)	1 (1.1)	3 (1.8)	7 (3.4)	1.29 (0.98-1.86)	0.11
Performed treatment						
Surgery	344 (39.8)	76 (39.8)	122 (40.3)	146 (39.5)	1.00 (0.94-1.06)	0.96
Elective^a^	67 (19.5)	19 (25.0)	21 (17.2)	27 (18.5)	0.97 (0.88-1.08)	0.60
Urgent^a^	223 (64.8)	44 (57.9)	81 (66.4)	98 (67.1)	1.04 (0.97-1.11)	0.28
Device extraction^a^	48 (14.0)	12 (15.8)	18 (14.8)	18 (12.3)	0.90 (0.77-1.04)	0.16
Urgent + device extraction^a^	6 (1.7)	1 (1.3)	2 (1.6)	3 (2.1)	1.08 (0.77-1.61)	0.69
Conservative treatment	402 (46.5)	102 (53.4)	138 (45.5)	162 (43.8)	0.94 (0.88-0.99)	0.023
Conservative despite indication	118 (13.7)	13 (6.8)	43 (14.2)	62 (16.8)	1.17 (1.07-1.29)	<0.001
High risk/frailty^a^	101 (85.6)	13 (100.0)	38 (88.4)	50 (80.6)	1.12 (1.02-1.24)	0.017
Patient’s wish^a^	17 (14.4)	0 (0.0)	5 (11.6)	12 (19.4)	1.48 (1.13-2.18)	0.015

Data are presented as n (%). “n” describes the number of patients in the subgroup. All models were adjusted for baseline characteristics, including infective endocarditis type (native valve or prosthetic) and the COVID-19 period. The results of the logistic regression analyses for change over time (diagnosis year) are shown as adjusted OR (aOR) with 95% CI, and corresponding *P* value.

a Percentage within group.

b *P* value for diagnosis year analyzed in the logistic regression model.

### Outcomes

**Figure 4:**
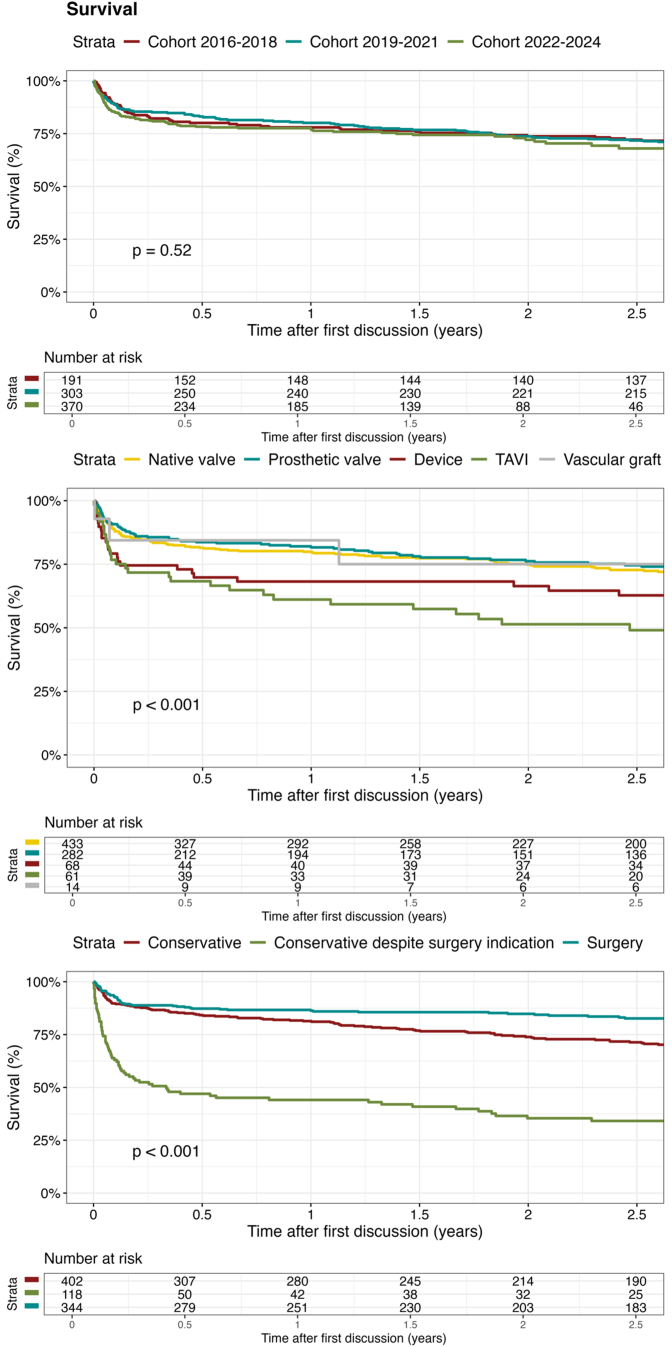
Kaplan-Meier Survival Curves for Patients With Possible/Definite infective endocarditis The upper Kaplan-Meier curve shows survival of the 3 cohorts. Cohort 1 (2016-2018) is shown in red, cohort 2 (2019-2021) in blue, and cohort 3 (2022-2024) in green. The middle Kaplan-Meier curve shows survival stratified for infection type: native valve endocarditis (yellow), prosthetic valve endocarditis (blue), transcatheter aortic valve endocarditis (green), cardiac implantable electronic device infection (red), or vascular graft infection (gray). The lower Kaplan-Meier curve shows survival stratified for treatment: conservative treatment (red), conservative treatment despite having an indication for surgery (green), and surgical treatment (blue). Abbreviation as in Figure 1.

**Figure 5:**
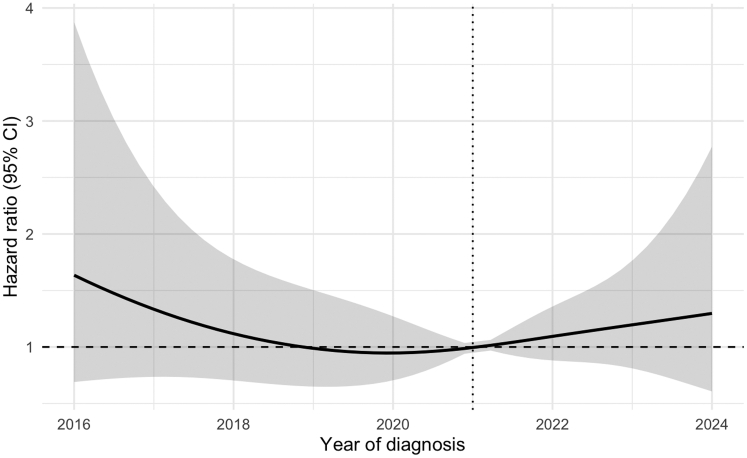
The HR for 1-Year Mortality Over Time The figure shows the adjusted HR (aHR) with 95% CI for 1-year mortality over time (2016-2024), with 2021 set as the reference year. The following covariates were included in the Cox model: age; medical history of COPD, heart failure, heart valve disease, hypertension, diabetes, chronic renal failure, stroke, and congenital heart disease; previous episode of endocarditis; and the COVID-19 period. The model was stratified for type of infection and type of treatment.

Follow-up completeness for survival among patients with possible/definite IE was 99.1%. The median [Q1-Q3] follow-up times for survival were 6.3 [1.3-7.3], 3.8 [1.8-4.8], and 0.9 [0.2-1.9] years for cohorts 1, 2, and 3, respectively. Survival was not significantly different between the cohorts, with a 1-year survival of 78.0%, 80.1%, and 77.2% for cohorts 1, 2, and 3, respectively (*P* = 0.62) (Figure 4). These observations were maintained after multivariable adjustment (aHR cohort 2 vs 1: 0.91; 95% CI: 0.66-1.25; *P* = 0.68, aHR cohort 3 vs 1: 0.92; 95% CI: 0.66-1.31; *P* = 0.97) (Supplemental Table 5). The HR for 1-year mortality per diagnosis year is visualized in Figure 5.

Survival was significantly different between types of IE (*P* < 0.001) (Figure 4). TAVI-IE had the highest mortality, with a 1-year survival of 61.1% compared to 80.0% and 81.8% for NVE and PVE (both *P* < 0.001). Patients operated on, treated conservatively without indication for surgery, and treated conservatively despite indication for surgery had a 1-year survival of 86.3%, 81.4%, and 44.1%, respectively. Survival was better for patients treated surgically compared to patients treated conservatively (*P* < 0.001). Survival was worse for patients treated conservatively despite having an indication for surgery compared to patients treated surgically or conservatively without indication for surgery (both *P* < 0.001).

**Figure 6:**
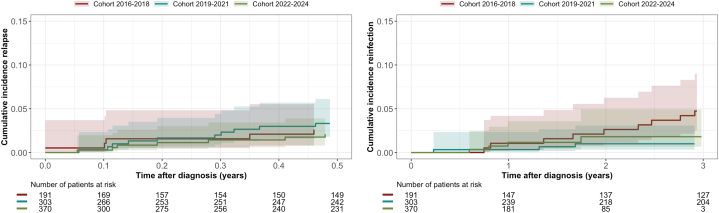
Cumulative Incidence Curves for Relapse and Reinfection The figure shows the cumulative incidence curves (including 95% CI) for relapse and reinfection in patients with definite/possible infective endocarditis, with death as a competing risk. The cumulative incidence curve for relapse is shown for the first 6 months after diagnosis and for relapse until 3 years after diagnosis. One patient had a reinfection with a different microorganism within 6 months after the initial diagnosis. Cohort 1 (2016-2018) is shown in red, cohort 2 (2019-2021) in blue, and cohort 3 (2022-2024) in green.

Relapse occurred in 23 (2.7%) and reinfection in 32 (3.7%) patients. The median [Q1-Q3] follow-up times for relapse and reinfection were 2.6 [1.3-3.8], 1.3 [0.1-2.8], and 0.5 [0.1-1.3] years for cohort 1, 2, and 3, respectively. The HR for relapse and reinfection was not significantly different over time (aHR relapse: 0.98; 95% CI: 0.83-1.02; *P* = 0.82, aHR reinfection: 0.99; 95% CI: 0.82-1.01; *P* = 0.98). Figure 6 shows the cumulative incidence of relapse and reinfection with death as a competing risk.

## Discussion

The aim of this study was to evaluate changes in the diagnosis, management, and outcome of patients with (suspected) IE, in the 9 years our ET has been installed (Central Illustration). The number of referrals and need for rediscussing increased. The need for advice for additional tests or change of treatment decreased over time, suggesting a learning effect of the ET. Nevertheless, the percentage of patients with additional diagnostic tests advised was still substantial in cohort 3 (40.4%). The type of advised test shifted from mainly microbiological to “other” tests, and indicates improved microbiological testing before referral. This may be partially explained by the publication of the national guideline paper: “Addendum Infectieuze Endocarditis”, authorized and published in 2020, which holds comprehensive information on adequate collection of initial blood cultures, as well as further microbiological testing in the case of (suspected) blood culture-negative IE.^11^ “Other” additional tests seem to have gained more attention in our ET, and are important to identify the portal of entry. Despite an overall decrease in the advice for additional tests, change in diagnosis or treatment after additional testing remained stable. This might indicate a learning effect of the ET for the referrers, or a more critical view on the expected benefit of additional tests, which is important in light of “choosing wisely”.^12^

**Central Illustration:**
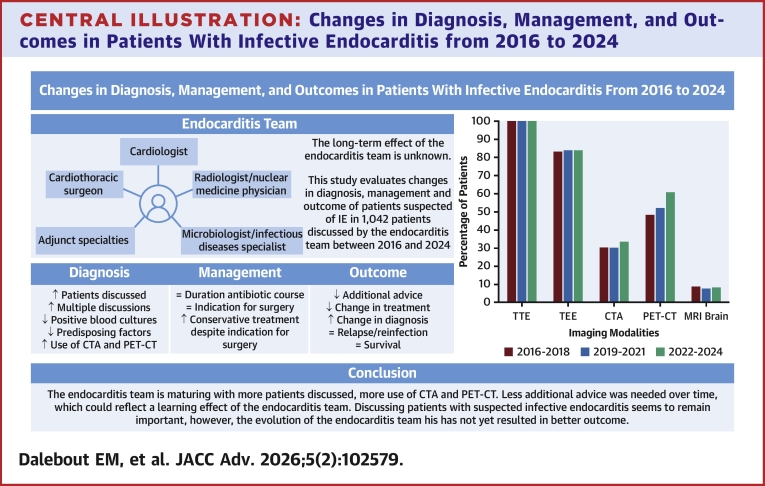
Changes in Diagnosis, Management, and Outcomes in Patients With Infective Endocarditis from 2016 to 2024 Evolution of diagnosis, management, and outcomes of patients with (suspected) infective endocarditis in the 9 years after introducing the endocarditis team. CTA = computed tomography angiography; IE = infective endocarditis; MRI = magnetic resonance imaging; PET-CT = positron emission tomography and computed tomography; TEE = transesophageal echocardiography; TTE = transthoracic echocardiography.

Overall, change of therapy following discussion by the ET significantly decreased, from 62.3% of patients in cohort 1 to 44.0% in cohort 3. Within this group, change in antibiotic therapy was advised in 40.3%, and decreased over time. This decrease is probably due to the update of the national “Stichting Werkgroep Antibioticabeleid” guidelines for the antimicrobial treatment of IE in 2019, which describes the antimicrobial regime for different causative pathogens and types of IE.^13^ This rate of change in antimicrobial management is in line with other studies, which reported a 35% to 90% change and improved adherence to appropriate antibiotic therapy after introducing the ET.^14^, ^15^, ^16^ In addition, Sadeghpour et al.^17^ found a lower complication rate after introducing the ET, which might be caused by improved antibiotic therapy. Therefore, an adjustment of the antimicrobial therapy remains important, and the additional value of the ET is still substantial with 31.2% change in diagnosis year 2024.

The overall change in diagnosis increased, even though the advice for additional tests decreased. This may be due to complete re-evaluation of the all available microbiological and imaging findings by an experienced ET.

The increasing number of referrals shows growing familiarity with the ET among referring doctors, since 72.7% of referrals were from other hospitals. It may also indicate an overall increase in the incidence or recognition of IE. Another contributing factor might be a lowered threshold for referral over the years, resulting in discussion of more patients with (suspected) IE. Our data support this, because patients in the later cohorts have less comorbidity and meet less diagnostic criteria. However, cases of uncomplicated IE might still not be referred to the ET, introducing a risk of referral, and thereby selection bias. Ideally, we would describe the total population of IE patients in our catchment area in this study. On the other hand, the estimated time expenditure of the ET is 10 to 15 hours per week, and can only be justified if it leads to improved management and outcome. Although initial diagnostic management improved, outcomes did not. Therefore, a critical question is if all patients referred to the ET required expert multidisciplinary care.

CTA and PET-CT were increasingly used for diagnostic imaging, although the percentage of scans positive for IE and the use of other diagnostic imaging tools were stable. This could be a result of the more prominent role of CTA and PET-CT in both NVE and PVE in the most recent 2023 ESC guidelines, which also recommends PET-CT for detection of peripheral lesions and portal of entry.^5^ Again, in light of “choosing wisely”, it remains important to consider the expected additional value. This is specifically important for PET-CT, as the scan is expensive, associated with radiation exposure and requires a low-carbohydrate diet and fasting period before the scan.^12^^,^^18^ The amount of CTA and PET-CT scans performed in other registries differs widely. The EURO-ENDO registry reported 65.9% CTA and 33.9% PET-CT scans performed in Western Europe, but recently published Dutch and Danish studies reported only 7% use of CTA and up to 67% use of PET-CT.^2^^,^^19^^,^^20^ The difference in use of these imaging modalities might differ between centers and countries due to differences in availability and expertise.

The ET discussion has an important role in surgical decision-making, as most referring centers do not have a cardiothoracic surgery department. The role of the ET lies in both decision-making, and timing of surgery. The surgical management changed over time, with an increasing percentage of patients treated conservatively despite having an indication for surgery. The main reason to decline surgery was frailty or comorbidity. However, the baseline characteristics of our cohorts showed similar age and a decrease in comorbidities. Despite this, 1-year survival was not significantly different between the 3 cohorts, but survival was significantly worse for patients with an indication for surgery that were treated conservatively. This is in line with findings in another study.^21^ Our results also show a small but significant advantage for the surgical compared to the conservative group. However, this is not a randomized controlled trial so we cannot draw any causal conclusions from this finding. Importantly, worse outcomes were observed for TAVI-IE patients. As TAVI-IE patients are more frail, suppressive antimicrobial therapy might be the choice of therapy for the patients that are not suitable for surgery, although evidence for suppressive antimicrobial therapy is still limited^22^, ^23^, ^24^, ^25^ and more studies are needed.

### Study Limitations

Due to the retrospective design of the study, missing data were inevitable; however, the amount of missing data is mostly minimal: Data on vegetation size are missing in most cases, with 41.6% of missing values, followed by the duration of antibiotic therapy, which was missing for 6.7% of the patients. Other variables had <2.0% missing data. We did not correct *P* values for multiple testing. Therefore, significance can be due to chance (type I error). Another limitation is that the referral pattern has changed. More patients from other centers were discussed over the years, and some important changes in baseline characteristics were observed such as a decrease in the number of patients with a prior history of endocarditis and prior valve surgery. In addition, selection bias is probably an issue in this study, since it is likely that more straightforward cases were diagnosed and treated in the local hospital without being discussed in our ET. The regional functioning of the ET also impedes the collection on recent outcome data, such as relapse and reinfection. We hypothesize that most patients previously discussed in our ET, would be discussed in our ET again in case relapse or reinfection occurs. However, rehospitalizations that occur outside of our institution and without referral to our ET would not be captured, potentially resulting in loss to follow-up and missingness. Moreover, a new European (ESC) guideline for the management of patients with IE was introduced within the study period. Changes in these guidelines, for example, the more prominent role of PET-CT, might have influenced diagnosis and management of patients in the different cohorts.^3^^,^^5^ Finally, we were not able to describe an historical cohort with patients with (suspected) IE before introduction of the ET, or a cohort with patients that were not discussed by our ET. Therefore, we cannot show the impact of the ET compared to patients that were not discussed by the ET.

### Clinical implications

The number of patients and need for rediscussions increased, increasing the time expenditure of the ET. An institutional or regional ET remains important for diagnosis and patient management, and appears to have an educational effect, because the need for additional diagnostic advice decreased. Therefore, an ET should be available all around Europe. However, the time expenditure is high and mortality remained stable, which warrants critical evaluation of the need for multidisciplinary consultation.

In terms of the setting up a regional ET, we found that clear arrangements concerning the core team and their time expenditure, timely registration and preparation, a good and safe working system to share images, and a clear and respectful manner to share recommendations are important. Our ET is held biweekly and online, which lowers the referral threshold and makes it easier for referrers to attend the discussion.

## Conclusions

The ET discussed more patients over time, and there was more need for rediscussions. The use of CTA and PET-CT imaging increased. Advice for additional tests and change in antibiotic treatment decreased, suggesting a learning effect and correct use of the existing guidelines. However, the impact of the ET remains important. Survival did not improve over time and mortality is still impressively high. Treatment was more often conservative despite having an indication for surgery, and this group had significantly worse survival.

## Funding support and author disclosures

Dr Budde received speakers fees from Bayer and Siemens and served on an advisory board for Bayer (all payments to Erasmus MC). Dr Swart has received consultancy fees from Amgen and Sanofi that are not relevant to the contents of this paper. Dr Hirsch received a research grant and consultancy fees from GE Healthcare and speaker fees from GE Healthcare, Bristol Myers Squibb, Bayer, Siemens Healthineers, and Heartflow; he is a member of the medical advisory board of Medis Medical Imaging Systems; and he was MRI corelab supervisor of Cardialysis BV until 2022. Institutional support to Erasmus MC by Siemens, Heartflow, and Bracco by research grants. All other authors have reported that they have no relationships relevant to the contents of this paper to disclose.

Perspectives **COMPETENCY IN MEDICAL KNOWLEDGE:** The findings of this study endorse the current guideline recommendation to discuss all patients with suspected IE in a multidisciplinary ET for early diagnosis, prevention of complications and improved and uniform treatment. In addition to advantages of an ET for patient care, we observed a learning effect for both the local specialists and the referring doctors involved in the ET discussions.

**TRANSLATIONAL OUTLOOK:** Setting up an ET can be challenging and also depends on local clinical processes and workflow. In our experience, a predefined date and time and a predefined dedicated team of ET members involved in the discussions in essential in setting up an ET. Furthermore, it is important to discuss secure digital options for the ET discussions and to transfer patient information, including imaging files. Clear arrangements should also be made on the communication of the ET advice to the referring doctor. In this study, we observed an increased use of CTA and PET-CT over time, although prognosis remains worrisome. This warrants the need for further optimization of indications to proceed to advanced imaging. Ideally, future studies would look more detailed into additional information gained by advanced imaging and associated changes in clinical management.
