# Supplementary material for: Evolution of Management and Outcomes of Infective Endocarditis After Introducing the Endocarditis Team
Source: JACC Adv. 2026 Jan 29;5(2):102579. doi: 10.1016/j.jacadv.2025.102579 (PMC12948601; doi:10.1016/j.jacadv.2025.102579)
Supplement: Supplementary Tables 1-5 [file mmc1.docx]

**Supplemental material**

Supplemental table 1…………………………………………………………page 2

Supplemental table 2…………………………………………………………page 4

Supplemental table 3…………………………………………………………page 6

Supplemental table 4…………………………………………………………page 7

Supplemental table 5…………………………………………………………page 8**Supplemental table 1** – Overview of missing data

| **Variable** | **Missing values (n)** | **Total (n)** | **Missing (%)** |
| --- | --- | --- | --- |
| Referring center | 0 | 1042 | 0.0 |
| Age (years) | 0 | 1042 | 0.0 |
| Sex | 0 | 1042 | 0.0 |
| Prior episode of endocarditis | 0 | 1042 | 0.0 |
| Pre-existent valve disease | 0 | 1042 | 0.0 |
| Congenital heart disease | 0 | 1042 | 0.0 |
| Prior heart valve surgery | 0 | 1042 | 0.0 |
| Prior device implantation | 0 | 1042 | 0.0 |
| Type of device in situ | 0 | 184 | 0.0 |
| Prior vascular prosthesis | 0 | 1042 | 0.0 |
| Type of vascular prosthesis in situ | 0 | 120 | 0.0 |
| Hypertension | 0 | 1042 | 0.0 |
| Heart failure | 0 | 1042 | 0.0 |
| Chronic renal failure | 0 | 1042 | 0.0 |
| COPD | 0 | 1042 | 0.0 |
| Diabetes mellitus | 0 | 1042 | 0.0 |
| Intravenous drug use | 0 | 172 | 0.0 |
| Positive blood cultures | 0 | 1042 | 0.0 |
| Causative pathogen | 0 | 1042 | 0.0 |
| Positive imaging | 0 | 1042 | 0.0 |
| TTE/TEE positive | 2 | 1042 | 0.2 |
| TEE performed | 2 | 1042 | 0.2 |
| TEE positive | 0 | 872 | 0.0 |
| CTA performed | 0 | 1042 | 0.0 |
| CTA positive | 0 | 331 | 0.0 |
| PET-CT performed | 0 | 1042 | 0.0 |
| PET-CT positive | 0 | 575 | 0.0 |
| Brain MRI performed | 0 | 1042 | 0.0 |
| Brain MRI positive | 0 | 87 | 0.0 |
| Predisposition | 0 | 1042 | 0.0 |
| Fever | 1 | 1042 | 0.1 |
| Embolic complications | 0 | 1042 | 0.0 |
| Cerebral embolization | 0 | 389 | 0.0 |
| Cerebral embolization at presentation | 0 | 133 | 0.0 |
| Other embolic complication | 0 | 389 | 0.0 |
| Other microbiological evidence | 0 | 1042 | 0.0 |
| Immunological phenomena | 0 | 1042 | 0.0 |
| Additional test advised | 0 | 1042 | 0.0 |
| Microbiological test advised | 0 | 1042 | 0.0 |
| Imaging test advised | 0 | 1042 | 0.0 |
| Other test advised | 0 | 1042 | 0.0 |
| Additional test changed therapy | 7 | 481 | 1.5 |
| Additional test changed therapy from conservative to invasive | 0 | 81 | 0.0 |
| Additional test changed antibiotic therapy | 0 | 81 | 0.0 |
| Additional test changed diagnosis | 7 | 481 | 1.5 |
| Change in therapy (overall) | 0 | 1042 | 0.0 |
| Change in diagnosis (overall) | 1 | 1042 | 0.1 |
| Final diagnosis | 0 | 1042 | 0.0 |
| Vegetation size | 360 | 864 | 41.6 |
| Severe valve regurgitation as a consequence of IE | 0 | 864 | 0.0 |
| Heart failure as a consequence of IE | 0 | 864 | 0.0 |
| Peri-annular complications | 0 | 864 | 0.0 |
| Type of aortic valve suspected for infection | 0 | 1042 | 0.0 |
| Type of mitral valve suspected for infection | 0 | 1042 | 0.0 |
| Type of pulmonary valve suspected for infection | 0 | 1042 | 0.0 |
| Type of tricuspid valve suspected for infection | 0 | 1042 | 0.0 |
| Device suspected for infection | 0 | 1042 | 0.0 |
| Type of device suspected for infection | 0 | 124 | 0.0 |
| Vascular prosthesis suspected for infection | 0 | 1042 | 0.0 |
| Type of vascular prosthesis suspected for infection | 0 | 93 | 0.0 |
| Indication for treatment | 0 | 864 | 0.0 |
| Duration of antibiotic therapy | 58 | 864 | 6.7 |
| Chronic suppressive therapy | 0 | 864 | 0.0 |
| Indication for elective surgery | 0 | 462 | 0.0 |
| Indication for urgent surgery | 0 | 462 | 0.0 |
| Indication for urgent surgery specified | 0 | 323 | 0.0 |
| Indication for device extraction | 0 | 462 | 0.0 |
| Performed treatment | 0 | 864 | 0.0 |
| Elective surgery performed | 0 | 344 | 0.0 |
| Urgent surgery performed | 0 | 344 | 0.0 |
| Device extraction performed | 0 | 344 | 0.0 |
| Reason surgery was not performed | 0 | 118 | 0.0 |
| CT: computed tomography; CTA: computed tomography angiography; IE: Infective endocarditis; MRI: magnetic resonance imaging; PET: positron emission tomography; TTE: transthoracic echocardiography; TEE: transesophageal echocardiography; | | | |

**Supplemental table 2** – Suspected location of infection. Within one patient, multiple locations of infection can be present.

| Location of suspected infection | Total  N = 1042 | 2016-2018  N = 223 | 2019-2021  N = 364 | 2022-2024  N = 455 | p-value |
| --- | --- | --- | --- | --- | --- |
| Type of aortic valve | | | | | |
| Native | 292 (28.0) | 61 (27.4) | 95 (26.1) | 136 (29.9) | 0.35 |
| Biological prosthesis | 143 (13.7) | 32 (14.3) | 47 (12.9) | 64 (14.1) |  |
| Mechanical prosthesis | 109 (10.5) | 28 (12.6) | 41 (11.3) | 40 (8.8) |  |
| Homograft | 5 (0.5) | 0 (0.0) | 0 (0.0) | 5 (1.1) |  |
| Autograft | 1 (0.1) | 0 (0.0) | 0 (0.0) | 1 (0.2) |  |
| Percutaneous prosthesis | 74 (7.1) | 17 (7.6) | 24 (6.6) | 33 (7.3) |  |
| Plasty | 2 (0.2) | 0 (0.0) | 0 (0.0) | 2 (0.4) |  |
| Type of mitral valve | | | | | |
| Native | 274 (26.3) | 54 (24.2) | 105 (28.8) | 115 (25.3) | 0.12 |
| Biological prosthesis | 8 (0.8) | 0 (0.0) | 7 (1.9) | 1 (0.2) |  |
| Mechanical prosthesis | 30 (2.9) | 10 (4.5) | 11 (3.0) | 9 (2.0) |  |
| Homograft | 0 (0.0) | 0 (0.0) | 0 (0.0) | 0 (0.0) |  |
| Autograft | 0 (0.0) | 0 (0.0) | 0 (0.0) | 0 (0.0) |  |
| Percutaneous prosthesis | 1 (0.1) | 0 (0.0) | 0 (0.0) | 1 (0.2) |  |
| Plasty | 23 (2.2) | 5 (2.2) | 10 (2.7) | 8 (1.8) |  |
| Type of pulmonary valve | | | | | |
| Native | 9 (0.9) | 1 (0.4) | 1 (0.3) | 7 (1.5) | 0.096 |
| Biological prosthesis | 0 (0.0) | 0 (0.0) | 0 (0.0) | 0 (0.0) |  |
| Mechanical prosthesis | 0 (0.0) | 0 (0.0) | 0 (0.0) | 0 (0.0) |  |
| Homograft | 15 (1.4) | 5 (2.2) | 5 (1.4) | 5 (1.1) |  |
| Autograft | 0 (0.0) | 0 (0.0) | 0 (0.0) | 0 (0.0) |  |
| Percutaneous prosthesis | 13 (1.2) | 3 (1.3) | 7 (1.9) | 3 (0.7) |  |
| Plasty | 1 (0.1) | 1 (0.4) | 0 (0.0) | 0 (0.0) |  |
| Type of tricuspid valve | | | | | |
| Native | 26 (2.5) | 4 (1.8) | 15 (4.1) | 7 (1.5) | 0.30 |
| Biological prosthesis | 0 (0.0) | 0 (0.0) | 0 (0.0) | 0 (0.0) |  |
| Mechanical prosthesis | 2 (0.2) | 0 (0.0) | 1 (0.3) | 1 (0.2) |  |
| Homograft | 0 (0.0) | 0 (0.0) | 0 (0.0) | 0 (0.0) |  |
| Autograft | 0 (0.0) | 0 (0.0) | 0 (0.0) | 0 (0.0) |  |
| Percutaneous prosthesis | 2 (0.2) | 0 (0.0) | 0 (0.0) | 2 (0.4) |  |
| Plasty | 1 (0.1) | 0 (0.0) | 0 (0.0) | 1 (0.2) |  |
| Type of native valve | **N=540** | **N = 107** | **N = 193** | **N = 240** |  |
| Aortic | 292 (54.1) | 61 (57.0) | 95 (49.2) | 136 (56.7) | 0.48 |
| Mitral | 274 (50.7) | 54 (50.5) | 105 (54.4) | 115 (47.9) | 0.38 |
| Pulmonary | 9 (1.7) | 1 (0.9) | 1 (0.5) | 7 (2.9) | 0.13 |
| Tricuspid | 26 (4.8) | 4 (3.7) | 15 (7.8) | 7 (2.9) | 0.059 |
| Type of prosthetic valve | **N=412** | **N = 96** | **N = 143** | **N = 173** |  |
| Aortic | 334 (80.9) | 77 (80.2) | 112 (77.8) | 145 (83.8) | 0.63 |
| Mitral | 62 (15.0) | 15 (15.6) | 28 (19.4) | 19 (11.0) | 0.082 |
| Pulmonary | 29 (7.0) | 9 (9.4) | 12 (8.3) | 8 (4.6) | 0.16 |
| Tricuspid | 5 (1.2) | 0 (0.0) | 1 (0.7) | 4 (2.3) | 0.30 |
| Type of device | | | | | |
| Pacemaker | 67 (6.4) | 17 (7.6) | 22 (6.0) | 28 (6.2) | 0.054 |
| ICD | 39 (3.7) | 15 (6.7) | 11 (3.0) | 13 (2.9) |  |
| CRT-P | 1 (0.1) | 0 (0.0) | 0 (0.0) | 1 (0.2) |  |
| CRT-D | 16 (1.5) | 0 (0.0) | 7 (1.9) | 9 (2.0) |  |
| Other | 1 (0.1) | 0 (0.0) | 0 (0.0) | 1 (0.2) |  |
| Vascular prosthesis | | | | | |
| Ascending aorta | 11 (1.1) | 0 (0.0) | 7 (1.9) | 4 (0.9) | 0.028 |
| Ascending aorta + other ^a^ | 5 (0.5) | 1 (0.4) | 1 (0.3) | 3 (0.7) |  |
| Bentall | 68 (6.5) | 18 (8.1) | 20 (5.5) | 30 (6.6) |  |
| Bentall + other ^a^ | 1 (0.1) | 0 (0.0) | 1 (0.3) | 0 (0.0) |  |
| Other ^a^ | 8 (0.8) | 1 (0.4) | 0 (0.0) | 7 (1.5) |  |
| Data are presented as n (%). “n” describes the number of patients in the subgroup. ^a^: other vascular graft (e.g., hemi-arch, thoracic endovascular aortic repair). ICD: implantable cardioverter defibrillator; CRTP: Cardiac resynchronization therapy with a pacemaker; CRTD: cardiac resynchronization therapy with a defibrillator. | | | | | |

**Supplemental table 3** – Type of infection in patients with definite/possible infective endocarditis.

|  | Total  N = 864 | 2016-2018  N = 191 | 2019-2021  N = 304 | 2022-2024  N = 369 | p-value |
| --- | --- | --- | --- | --- | --- |
| Type of infection | | | | | |
| Native valve | 429 (49.7) | 89 (46.6) | 150 (49.5) | 190 (51.4) | 0.77 |
| Prosthetic valve | 284 (32.9) | 66 (34.6) | 103 (34.0) | 115 (31.1) |  |
| TAVI | 61 (7.1) | 14 (7.3) | 21 (6.9) | 26 (7.0) |  |
| Device | 70 (8.1) | 20 (10.5) | 23 (7.6) | 27 (7.3) |  |
| Vascular graft | 14 (1.6) | 1 (0.5) | 5 (1.7) | 8 (2.2) |  |
| Other | 6 (0.6) | 1 (0.5) | 1 (0.3) | 4 (1.1) |  |
| Percentage of patients with definite/possible infective endocarditis with infection of the native valve, prosthetic valve, cardiac implantable electronic device infection, transcatheter aortic valve (TAVI), vascular graft, or other location of infection. “Other” infection was present in 6 patients and included 2 patients with an Amplatzer device, 1 patient with abandoned ICD leads, 1 patient with a stent in the subclavian artery, 1 patient with an mycotic aneurysm of the right coronary artery and 1 patient with a transannular patch in the right ventricular outflow tract and ventricular septal defect closure with a patch. Data are presented as n (%).“n” describes the number of patients in the subgroup. | | | | | |

**Supplemental table 4** – Indication for urgent surgery for patients with definite/possible infective endocarditis.

|  | Total  N = 323 | 2016-2018  N = 60 | 2019-2021  N = 119 | 2022-2024  N = 144 | p-value |
| --- | --- | --- | --- | --- | --- |
| Indication for urgent surgery | | | | | |
| Congestive heart failure | 92 (28.5) | 16 (26.7) | 37 (31.1) | 39 (27.1) | 0.73 |
| Uncontrolled infection | 100 (31.0) | 19 (31.7) | 35 (29.4) | 46 (31.9) | 0.90 |
| Embolization | 67 (20.7) | 15 (25.0) | 20 (16.8) | 32 (22.2) | 0.37 |
| Perivalvular abscess/mycotic aneurysm | 133 (41.2) | 18 (30.0) | 55 (46.2) | 60 (41.7) | 0.11 |
| Other | 21 (6.5) | 4 (6.7) | 11 (9.2) | 6 (4.2) | 0.26 |
| More than one indication for urgent surgery can be applicable for the same patient. Data are presented as n (%). “n” describes the number of patients in the subgroup. | | | | | |

**Supplemental table 5** – The univariable and multivariable Cox proportional hazard model(s) for all-cause mortality.

|  | **Univariable** | | **Multivariable** | |
| --- | --- | --- | --- | --- |
| **Term** | **HR (95%CI)** | **p-value** | **HR (95%CI)** | **p-value** |
| Cohort 2 (reference: Cohort 1) | 1.11 (0.82-1.50) | 0.49 | 1.08 (0.74-1.59) | 0.69 |
| Cohort 3 (reference: Cohort 1) | 1.21 (0.87-1.68) | 0.25 | 1.01 (0.71-1.43) | 0.97 |
| Sex | 1.44 (1.13-1.84) | 0.003 | 1.28 (0.99-1.66) | 0.061 |
| Age/10y | 1.05 (1.04-1.06) | <0.001 | 1.44 (1.29-1.61) | <0.001 |
| Hypertension | 2.19 (1.74-2.76) | <0.001 | 1.48 (1.14-1.93) | 0.003 |
| Diabetes | 1.79 (1.38-2.32) | <0.001 | 1.12 (0.84-1.49) | 0.43 |
| COPD | 1.58 (1.10-2.26) | 0.014 | 1.11 (0.75-1.64) | 0.61 |
| Heart Failure | 1.77 (1.33-2.37) | <0.001 | 1.40 (1.01-1.93) | 0.045 |
| Heart valve disease | 1.28 (1.02-1.61) | 0.037 | 0.81 (0.60-1.09) | 0.17 |
| Prior episode of endocarditis | 0.93 (0.58-1.48) | 0.67 | 0.99 (0.58-1.68) | 0.97 |
| Chronic renal failure | 2.73 (2.04-3.65) | <0.001 | 2.16 (1.57-2.96) | <0.001 |
| Covid period | 1.03 (0.79-1.35) | 0.82 | 0.77 (0.53-1.13) | 0.19 |

Proportional hazard assumption was violated for the type of infection and type (native valve endocarditis, prosthetic valve endocarditis, transcatheter aortic valve endocarditis, cardiovascular implantable electronic device infection, vascular graft infection, or other) and of treatment (conservative, surgery, conservative despite indication for surgery) and the cox model is stratified for these variables, as such no covariates of these variables are presented.
